# Hexanoic Acid Intake Enhances Anti‐Tumor Immune Responses in Colorectal Cancer by Reducing the Immunosuppressive Function of Tregs

**DOI:** 10.1002/mnfr.70229

**Published:** 2025-08-23

**Authors:** Daichi Seta, Kenta Nagahori, Yuki Katoh, Tadashi Ogawa, Takashi Iwata, Hiroshi Nishio, Masaki Sugawara, Yosuke Fujii, Ayano Yoshido, Hiroshi Seno, Shuichi Hirai

**Affiliations:** ^1^ Division of Anatomical Science Department of Functional Morphology Nihon University School of Medicine Itabashi‐ku Tokyo Japan; ^2^ Department of Anatomy Tokai University School of Medicine Isehara Kanagawa Japan; ^3^ Department of Obstetrics and Gynecology Keio University School of Medicine Shinjuku‐ku Tokyo Japan; ^4^ Department of Legal Medicine Aichi Medical University School of Medicine Nagakute Aichi Japan

**Keywords:** anti‐tumor immune response, colorectal cancer, hexanoic acid, milk, regulatory T cell

## Abstract

Colorectal cancer (CRC) remains a leading cause of cancer mortality worldwide. Regulatory T cells (Tregs) are key immune regulators that inhibit anti‐tumor immune responses by suppressing effector T‐cell functions and fostering an immunosuppressive tumor microenvironment. Despite epidemiological evidence showing an inverse association between milk consumption and CRC risk, the underlying mechanisms remain unclear. While milk‐derived fatty acids have demonstrated tumor‐suppressive activities, the anti‐tumor effects of hexanoic acid, a characteristic component of milk fat, have not been thoroughly investigated. Model tumor mice orally administered hexanoate, the sodium salt of hexanoic acid, showed increased hexanoic acid concentrations in tumor‐draining lymph nodes and tumor sites, with enhanced anti‐tumor immune responses that significantly suppressed cancer cell growth. These effects were mediated through the following: (1) suppressed differentiation of naïve CD4^+^ T cells into Tregs; (2) decreased Treg‐mediated inhibition of CD8^+^ T cells; and (3) suppressed intratumoral infiltration of Tregs, indirectly enhancing the effector function of CD8^+^ T cells. Furthermore, hexanoate enhanced the therapeutic effect of immune checkpoint inhibition therapy. Part of the protective effect of milk intake against CRC development may be mediated by hexanoic acid, which enhances anti‐tumor immune responses through its actions on Tregs.

## Graphical abstract

This study found hexanoic acid (HA), a fatty acid in milk, inhibits colorectal cancer by enhancing anti‐tumor immune responses. HA suppresses naïve CD4+ T cell differentiation into Tregs, reduces the immunosuppressive activity of Tregs against CD8+ T cells, and decreases tumor infiltration of Tregs. These mechanisms indirectly enhance CD8+ T cell anti‐tumor activity. HA also enhances anti‐PD‐1 antibody therapy, showing promise as a dietary supplement for improving cancer immunotherapy.

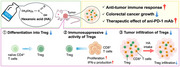

**Abbreviations**
ACT: adoptive cell therapy
APC: allophycocyanin
BV: Brilliant Violet
CAR‐T: chimeric antigen receptor T‐cell
CFSE: carboxyfluorescein diacetate succinimidyl ester
CRC: colorectal cancer
FBS: fetal bovine serum
ICI: immune checkpoint inhibitor
IHC: immunohistochemistry
MACS: magnetic‐activated cell sorting
MDSCs: myeloid‐derived suppressor cells
PD‐1: programmed death‐1
PE: phycoerythrin
TAMs: tumor‐associated macrophages
Th: T helper
TILs: tumor‐infiltrating lymphocytes
TME: tumor microenvironment
Tregs: regulatory T cells

## 1 Introduction

Colorectal cancer (CRC) accounts for approximately 10% of all annually diagnosed cancers and cancer‐related deaths worldwide and is the world's fourth most deadly cancer, with almost 900 000 deaths annually [1, 2]. The global incidence of CRC is predicted to increase to 2–5 million cases in 2035 [2]. The development of CRC involves a variety of environmental factors, among which diet is a major contributor [3]. Many epidemiological studies have reported an inverse association between milk intake and risk of death in CRC, indicating that milk may have a protective effect against CRC [4, 5, 6, 7]. Furthermore, multiple reports have indicated that the calcium in milk contributes to this effect by inhibiting CRC [8, 9]. However, another study observed a protective association between milk intake and CRC mortality that appeared to be independent of calcium intake [10]. Thus, calcium‐independent mechanisms for the effects of milk intake on cancer, especially those involving fatty acids abundant in milk, are attracting attention [11, 12].

SCFAs and medium‐chain fatty acids (MCFAs) are characteristic components of milk, each containing high percentages of butyric acid (C4:0), hexanoic acid (C6:0), decanoic acid (C10:0), and lauric acid (C12:0) [13, 14]. Recent studies have provided evidence to suggest that these milk‐derived fatty acids have anti‐cancer activity. Decanoic acid and lauric acid have been shown to suppress tumor growth in a variety of cancer types, including breast, skin, liver, and colon cancer, both in vitro and in vivo [15, 16, 17, 18]. Interestingly, butyric acid was demonstrated to enhance the anti‐tumor activity of CD8^+^ T cells in the tumor microenvironment (TME) of model mice [19]. Studies of chronic inflammatory diseases, such as Crohn's disease, have speculated that hexanoic acid suppresses cancer growth by modulating the immune system, upregulating inflammatory cytokine production, and promoting differentiation of naïve T cells into inflammatory T cells [20, 21]. However, the possibility that hexanoic acid positively regulates anti‐tumor immune responses to suppress tumor growth has not been tested.

In this study, we investigated the inhibitory effects of hexanoic acid on CRC in tumor‐bearing mice and examined its impact on anti‐tumor immune responses by characterizing immune cell types in the TME.

## 2 Experimental Section

### 2.1 Animals and Cell Culture

BALB/c mice (age: 6–8 weeks; CLEA Japan, Tokyo, Japan) were bred at the animal facilities of Nihon University, in accordance with its guidelines for animal experimentation. Mice were maintained in a specific‐pathogen‐free environment on a 12‐h light–dark cycle, with the dark cycle occurring from 8:00 p.m. to 8:00 a.m.

The mouse colorectal carcinoma cell line CT26 was purchased from the American Type Culture Collection (ATCC) and cultured in RPMI 1640 (Thermo Fisher Scientific, Waltham, MA, USA) containing 10% heat‐inactivated fetal bovine serum (FBS), 100 U/mL penicillin, and 100 µg/mL streptomycin at 37°C with 5% CO_2_ under 100% humidity. Before use in experiments, CT26 cells were recovered from liquid nitrogen, cultured for at least five passages, and verified to be negative for mycoplasma contamination using a TaKaRa PCR Mycoplasma Detection Set (Takara Bio Inc., Shiga, Japan).

Mouse T cells were cultured in RPMI 1640 (Thermo Fisher Scientific) containing 10% heat‐inactivated FBS, 100 U/mL penicillin, 100 µg/mL streptomycin and 500 IU/mL recombinant human IL‐2 (Novartis, Basel, Switzerland).

### 2.2 Tumor‐Bearing Mouse Modeling

Six‐ to 8‐week‐old BALB/c mice were inoculated subcutaneously in the flank with 5 × 10^5^ CT26 cells on day 0. Starting on day 4, mice were treated with vehicle (200 µL water) or 1 M sodium hexanoate in 200 µL water, twice daily for the experimental period. Mice in the combined therapy group also received anti‐programmed death‐1 (PD‐1) or isotype‐matched antibodies (200 µg/animal; Bio X Cell, Lebanon, NH, USA) on days 4, 7, and 10. For cell depletion, anti‐CD8 or isotype antibodies (200 µg/animal; Bio X Cell) were given intraperitoneally on days 1, 4, 5, 6, and 10. Tumor volume was calculated by direct tumor measurements every 4 days, using the following formula: 1/2 [length × (width)^2^]. The mouse cohorts used in each experiment are summarized in Figure S1.

### 2.3 Histological Analysis of Normal Mouse Tissue

Euthanized mice underwent transcardiac perfusion fixation, first with chilled PBS followed by chilled 10% neutral buffered formalin. Approximately 30 min following perfusion, livers, small intestines, colons, and brains were removed and immersion‐fixed in 10% neutral buffered formalin for 24 h at room temperature. Fixed tissues were processed using automatic processing and embedding equipment (Tissue TekVIP; Sakura Finetek Japan, Tokyo, Japan) through graded ethanol dehydration, xylene clearing, and paraffin embedding. Sections were cut at 4‐µm‐thickness, dewaxed in xylene, and rehydrated through a gradient ethanol series (100% to 30%) for 2 min at each step. After hematoxylin and eosin (HE) staining and xylene dehydration, the sections were mounted with permount (Falma, Tokyo, Japan) and observed under an Evident BX53 microscope (Evident, Tokyo, Japan).

### 2.4 Hexanoate Dosage Information

Mice received oral gavage of 200 µL water or 1 M sodium hexanoate (Sigma‐Aldrich, St. Louis, MO, USA) in 200 µL water starting on day 4 of modeling, twice daily for the experimental period. The dosage of this mouse model, equivalent to 2760 mg/kg/day, was determined with reference to previously described protocols [22, 23, 24]. No severe adverse effects, including weight loss and histological abnormalities, were observed with oral gavage of sodium hexanoate in this study (Figure S2a,b). The human equivalent dose of sodium hexanoate was calculated to be 165.6 g/day for a 60 kg human.

### 2.5 Samples and Sample Preparation for LC‐MS/MS

Draining lymph node cells (1 × 10^6^) and tumor tissue samples were ground using a multibead shocker (µT‐12; TAITEC, Saitama, Japan) with a frozen disruption device, and 600 µL methanol containing internal standard (10 µg hexanoic acid‐d11) was added. After the addition of 300 µL deionized water and 500 µL acetonitrile, the mixture was centrifuged at 20 000 × *g* for 15 min at 4°C. The liquid layer was collected and dried under nitrogen stream. Each sample was derivatized with 2‐picolylamine using 20 µL of 10 mM triphenylphosphine in acetonitrile, 20 µL of 10 mM 2,2′‐dipyridyl disulfide in acetonitrile and 20 µL of 2‐picolylamine (10 µg/µL) in acetonitrile. The derivatization was performed at 60°C for 10 min. For the analysis, the product was dissolved in the mobile phase (130 µL), 1 µL of which was subjected to LC‐MS/MS.

### 2.6 LC‐MS/MS Analyses

LC‐MS/MS analyses were performed on a Nexera X3 and LCMS‐8045 system (Shimazu, Kyoto, Japan). For separation, a Kinetex C8 column (2.1 × 100 mm, 2.6 µm; Phenomenex, Torrance, CA, USA) was used at 40°C. The mobile phase consisted of a gradient of solvent A (an aqueous solution of 0.1% formic acid and 10 mM ammonium formate) and solvent B (a methanolic solution of 0.1% formic acid and 10 mM ammonium formate) delivered at 0.3 mL/min. The composition of the mobile phase began at 40% B, was followed by 40%–55% B (0–6.0 min), 55%–98% B (6.0–7.0 min), and 98% B (7.0–12.0 min), and was held at 40% B for 2.0 min to equilibrate the column for the following sample.

The MS/MS was operated with an electrospray ionization source in the positive ionization mode. Ionization source conditions were as follows: nebulizer gas flow rate, 3.0 L/min; drying gas flow rate, 10 L/min; desolvation line temperature, 250°C; heating block temperature, 400°C; and collision gas pressure, 230 kPa. High‐purity argon and nitrogen were used as collision and nebulizer gases, respectively. Quantification was performed using selective reaction monitoring based on the peak area. The transition ions of hexanoic acid and hexanoic acid‐d11 were *m*/*z* 207.1 > 109.1 and *m*/*z* 218.2 > 109.1, respectively. Data acquisition and processing were performed using LabSolutions software (v 5.123; Shimadzu, Kyoto, Japan).

### 2.7 Flow Cytometry

For gp70‐specific CD8^+^ T‐cell staining, cells were stained with FITC‐conjugated anti‐CD8 (MBL, Nagoya, Japan), H‐2Ld MuLV gp70 tetramer, phycoerythrin (PE)‐labeled H‐2Ld β‐galactosidase tetramer (MBL), Brilliant Violet (BV)421‐conjugated CD3 (BioLegend, San Diego, CA, USA) and BV510‐conjugated CD45 (BD Biosciences, San Jose, CA, USA). Tumor‐infiltrating CD8^+^ T cells (CD8^+^ TILs) were stained with the following antibodies: PE/Dazzle 594‐conjugated anti‐CD4 (BioLegend), Alexa Fluor 700 (Alexa700)‐conjugated CD8 (BD Biosciences), allophycocyanin (APC)/Cy7‐conjugated CD3 (BioLegend), BV421‐conjugated PD‐1 (BioLegend), and BV510‐conjugated CD45 (BD Biosciences). Tumor‐associated macrophages (TAMs) and myeloid‐derived suppressor cells (MDSCs), were stained with APC‐conjugated anti‐Ly‐6G/Ly‐6C (Gr‐1) (BioLegend), Alex700‐conjugated F4/80 (BioLegend), BV421‐conjugated CD11b (BioLegend), and BV510‐conjugated CD45 (BD Biosciences). Tregs were stained with PE‐conjugated anti‐Foxp3 (BD Biosciences), PE/Dazzle 594‐conjugated CD4 (BioLegend), APC/Cy7‐conjugated CD25 (BioLegend), Alexa700‐conjugated CD3 (BioLegend), and BV510‐conjugated CD45 (BD Biosciences). Intracellular staining of Foxp3 was performed using Foxp3 Fix/Perm Buffer (BioLegend), in accordance with the manufacturer's instructions. Naïve, stem cell memory, central memory, and effector CD4^+^ T cells were stained with the following antibodies: FITC‐conjugated anti‐CD8 (MBL), PE‐conjugated anti‐CD45 (BioLegend), PE/Dazzle 594‐conjugated anti‐CD4 (BioLegend), PerCP/Cyanine (PC)5.5‐conjugated CD44 (BD Biosciences), APC‐conjugated anti‐ Ly‐6A/E (Sca‐1) (BioLegend), APC)/Cy7‐conjugated CD3 (BioLegend), and BV510‐conjugated CD62L (BD Biosciences).

Flow cytometry samples were acquired using a Gallios flow cytometer (Beckman Coulter, Brea, CA, USA) and analyzed using Kaluza software (Beckman Coulter).

### 2.8 Immunohistochemistry (IHC)

Tumor samples were obtained from mice 17 days after tumor inoculation. Formalin‐fixed, paraffin‐embedded sections of mouse tissues were subjected to IHC analysis, as described previously [25]. The CD8α (D4W2Z) XP rabbit mAb (mouse‐specific) was purchased from Cell Signaling. Histofine simple stain MAX‐PO (Nichirei Biosciences Inc., Tokyo, Japan) was used to detect primary antibodies in mouse tissues, in accordance with the manufacturer's instructions. CD8‐stained slides were scanned using a high‐resolution digital slide scanner (NanoZoomer‐XR C12000; Hamamatsu Photonics, Hamamatsu, Shizuoka, Japan). The number of CD8^+^ T‐cell infiltrates per 1 mm^2^ of tumor was calculated automatically using a computerized image analysis system (Tissue Studio; Definiens, Munich, Germany).

### 2.9 In Vitro Functional Analysis of CD8^+^ T Cells

CD8^+^ T cells were isolated from mice splenocytes using magnetic‐activated cell sorting (MACS). CD8^+^ T cells were cultured for 2 days in RPMI medium containing 10% serum, supplemented with or without 100 µM hexanoate. On day 2, CD8^+^ T cells were activated by stimulation with anti‐CD3 (clone 145‐2C11, 2 mg/mL) and anti‐CD28 (clone PV‐1, 2 mg/mL) mAbs and IL‐2. On day 5, cells and culture supernatants were collected and evaluated for cell proliferative capacity using a WST‐1 assay (Roche, Basel, Switzerland) and IFN‐γ production capacity using an ELISA (BD Biosciences Pharmingen, San Jose, CA, USA).

### 2.10 Differentiation of Mouse Naïve CD4^+^ T Cells Into Tregs or T Helper (Th)1 Cells

Mouse naïve CD4^+^ T cells were differentiated into Tregs or Th1 cells as described in previous reports [21]. Briefly, naïve CD4^+^ T cells (CD3^+^CD4^+^CD62L^+^CD44^low^) were isolated from mice splenocytes using a naïve CD4^+^ T‐cell isolation kit (Miltenyi Biotec, Bergisch Gladbach, Germany), in accordance with the manufacturer's instructions. For the differentiation of Tregs, sorted naïve CD4^+^ T cells were stimulated with anti‐CD3 mAb (2 mg/mL) and anti‐CD28 mAb (2 mg/mL) in the presence of recombinant human transforming growth factor‐β1 (1 ng/mL). For the differentiation of Th1 cells, anti‐CD3 and anti‐CD28 mAbs (2 mg/mL each), IL‐12 (20 ng/mL), and anti‐IL‐4 (10 mg/mL) were added to the culture medium. To determine its influence on naïve CD4^+^ T‐cell differentiation, cells were cultured with and without 250 µM hexanoate.

### 2.11 Treg Suppression Assay

The Treg suppression assay was performed as previously described [26]. Briefly, Tregs were isolated from tumors of mice treated with vehicle (vehi‐Tregs) or hexanoate (hexa‐Tregs) using a CD4^+^CD25^+^ Treg isolation kit (Miltenyi Biotec), in accordance with the manufacturer's instructions. To evaluate the suppression of CD8^+^ T‐cell proliferation, CD8^+^ T cells isolated from mice splenocytes were labeled with carboxyfluorescein diacetate succinimidyl ester (CFSE) using the CellTrace CFSE cell proliferation kit, in accordance with the manufacturer's instructions. CFSE‐labeled CD8^+^ T cells (1 × 10^5^) were co‐cultured with vehicle‐Tregs or hexa‐Tregs (1 × 10^4^) in the presence of anti‐CD3 mAb, anti‐CD28 mAb, and IL‐2. Cell proliferation was assessed 3 days later using flow cytometry.

To evaluate the suppression of IFN‐γ production by CD8^+^ T cells, 1 × 10^5^ CD8^+^ T cells isolated from mice splenocytes were co‐cultured with vehi‐Tregs or hexa‐Tregs at different ratios (Treg:CD8 at 0.1:1, 0.3:1, 1:1) in the presence of anti‐CD3 mAb, anti‐CD28 mAb and IL‐2. On day 3, culture supernatants were collected for ELISA measurement of IFN‐γ (BD Biosciences Pharmingen).

### 2.12 Statistical Analysis

Statistical analyses were performed using GraphPad Prism 10 software (version 4.0). Comparisons between two groups were made using unpaired two‐tailed Student's *t*‐tests. Multiple comparisons were made by one‐way analysis of variance with Tukey's multiple comparisons test. Data are presented as means ± SD. A *p* value < 0.05 was considered to indicate statistical significance.

## 3 Results

### 3.1 Hexanoate Intake Enhances Anti‐Tumor Immune Responses and Improves the Therapeutic Effect of Anti‐PD‐1 Antibody

**FIGURE 1:**
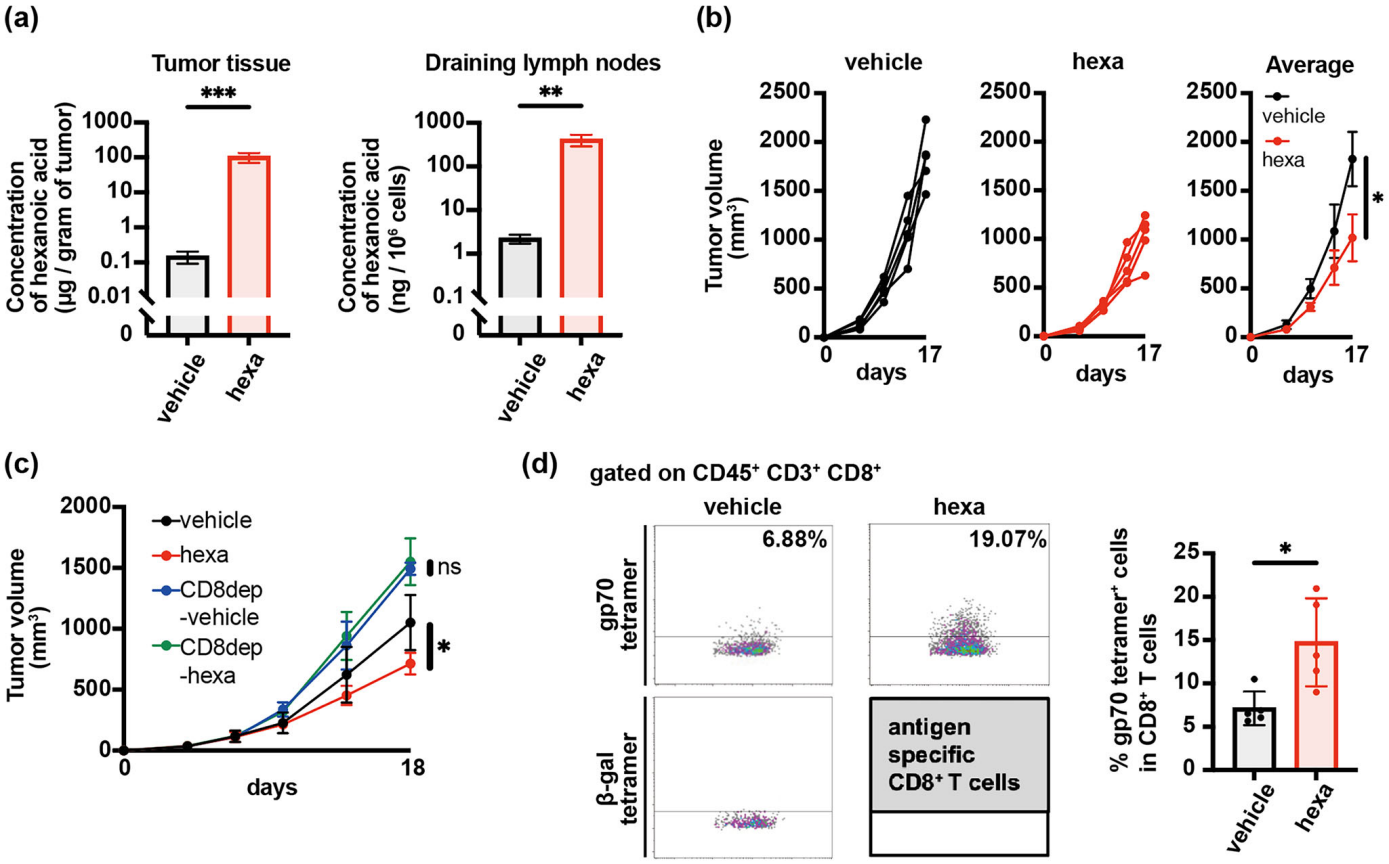
Hexanoate intake suppresses tumor growth by enhancing anti‐tumor immune responses. (a) Concentrations of hexanoic acid in CT26 tumors and draining lymph nodes (a), and tumor‐growth curves in individual BALB/c mice (left two panels) and average tumor volumes (mm^3^ ± SD; *n* = 5) (right panels) (b) following treatment with 1 M hexanoate (hexa; equivalent to 2760 mg/kg/day) or vehicle only (vehicle). (c) Average tumor volumes (mm^3^ ± SD; *n* = 5) in vehicle‐ and hexa‐treated mice that also received CD8^+^ T cell‐depleting or isotype‐matched mAbs. (d) Flow cytometry of gp70‐specific CD8^+^ T cells in tumors. Representative gp70‐tetramer staining of CD8^+^ T cells in each group (left panels) and analysis of all individuals (right panel) (mm^3^ ± SD; *n* = 5). Values are expressed as mean ± SD of different mice per group. Comparisons between two groups and multiple comparisons were performed using unpaired two‐tailed Student's *t*‐tests and one‐way analysis of variance with Tukey's multiple comparisons test, respectively. *p < 0.05, **p < 0.01, ***p < 0.001. n.s., not significant; Dep, depleted.

We evaluated the immunologic anti‐tumor effects and mechanism of hexanoate intake using BALB/c mice implanted with CT26 colon cancer cells. Oral administration of hexanoate, an SCFA/MCFA, increased the concentration of hexanoic acid in the tumor tissue and draining lymph nodes (Figure 1a), and significantly inhibited tumor growth (Figure 1b), in CT26 tumor‐bearing mice. To determine the cellular mechanism underlying this anti‐tumor effect, we depleted CD8^+^ T cells using specific antibodies. This depletion completely eliminated the hexanoate‐mediated inhibition of tumor growth (Figures 1c and S3), demonstrating the involvement of CD8^+^ T cells in the anti‐tumor effects of hexanoate in vivo. Staining of gp70/H‐2Ld‐tetramers revealed that hexanoate intake also significantly enhanced the induction of tumor antigen‐specific CD8^+^ T cells in tumors of CT26‐bearing mice (Figure 1d). These results showed that the anti‐tumor effect of hexanoate is elicited via an immune response through CD8^+^ T cells.

**FIGURE 2:**
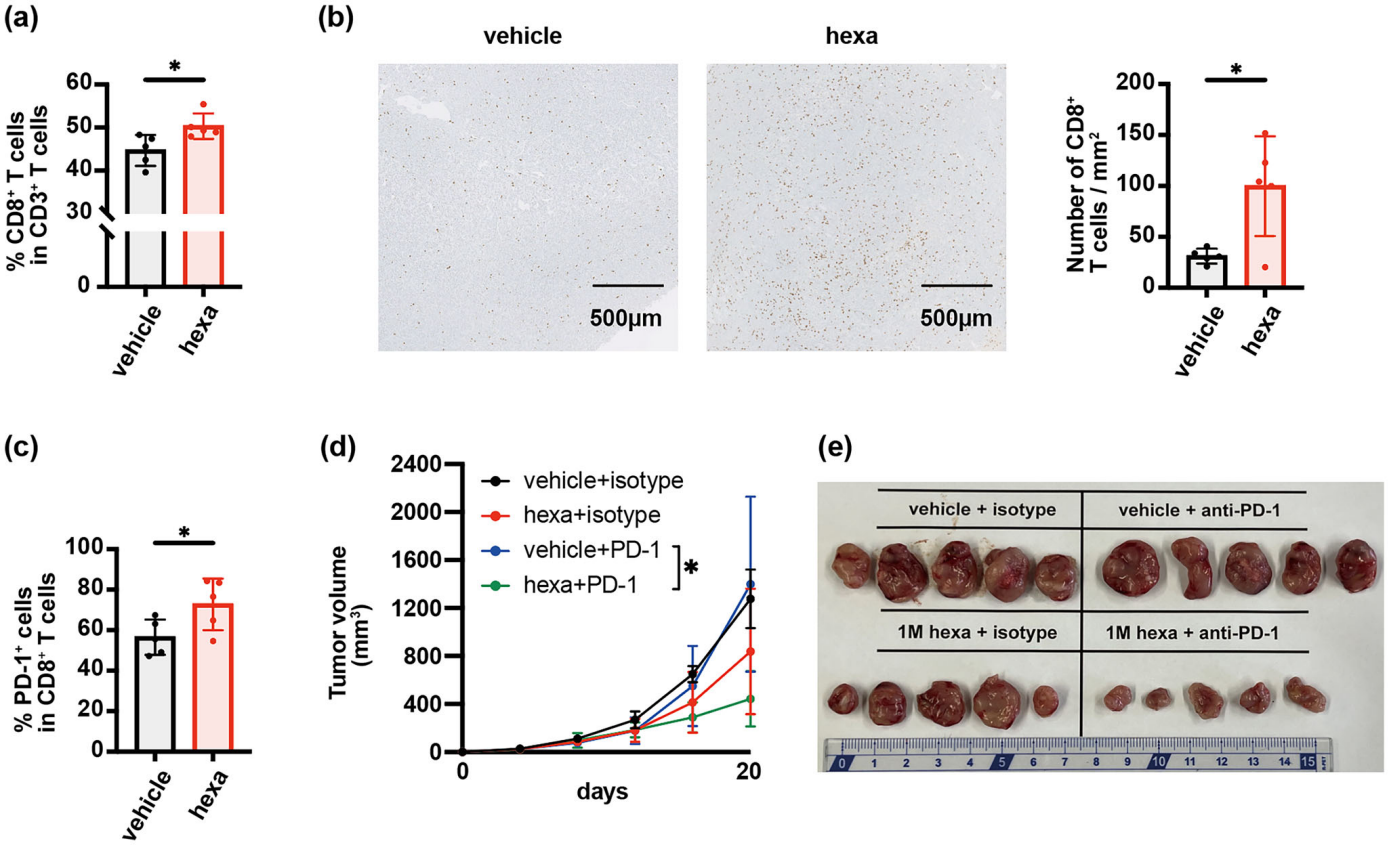
Hexanoate intake enhances CD8^+^ T‐cell intratumor infiltration and potentiates the therapeutic effect of anti‐PD‐1 antibodies. (a–c) Flow cytometry analysis of percentages of tumor‐infiltrating CD8^+^ T cells (a) with image analysis of immunostaining (b), and flow cytometry analysis of percentages of PD‐1^+^CD8^+^ T cells in CT26 tumors (c) of BALB/c mice (mm^3^ ± SD; *n* = 5) following treatment with 1 M hexanoate (hexa; equivalent to 2760 mg/kg/day) or vehicle only (vehicle). (d and e) Average tumor growth curves (d) and representative photographs (e) of CT26 tumors removed from mice treated with hexanoate or vehicle plus 200 µg/mouse of anti‐PD‐1 or isotype‐matched mAbs (means ± SD; *n* = 5). Values are expressed as mean ± SD of different mice per group. Comparisons between two groups and multiple comparisons were performed using unpaired two‐tailed Student's *t*‐tests and one‐way analysis of variance with Tukey's multiple comparisons test, respectively. *p < 0.05.

Flow cytometry and immunohistochemical analyses demonstrated that hexanoate administration increased the infiltration of CD8^+^ T cells into tumors (Figure 2a,b). It also enhanced the expression of PD‐1 in CD8^+^ TILs, suggesting the activation and exhaustion of tumor antigen‐specific T cells in tumors (Figure 2c). Given the increased expression of PD‐1 in CD8^+^ TILs and induction of antigen‐specific T cells, we evaluated the therapeutic effect of hexanoate in combination with anti‐PD‐1 antibodies. Hexanoate enhanced the therapeutic efficacy of anti‐PD‐1 antibodies (Figures 2d,e and S4), strongly suggesting that it potentiates this immunotherapy by promoting anti‐tumor responses within the TME.

### 3.2 Systemic Administration of Hexanoate Suppresses the Number of Intratumoral Tregs

**FIGURE 3:**
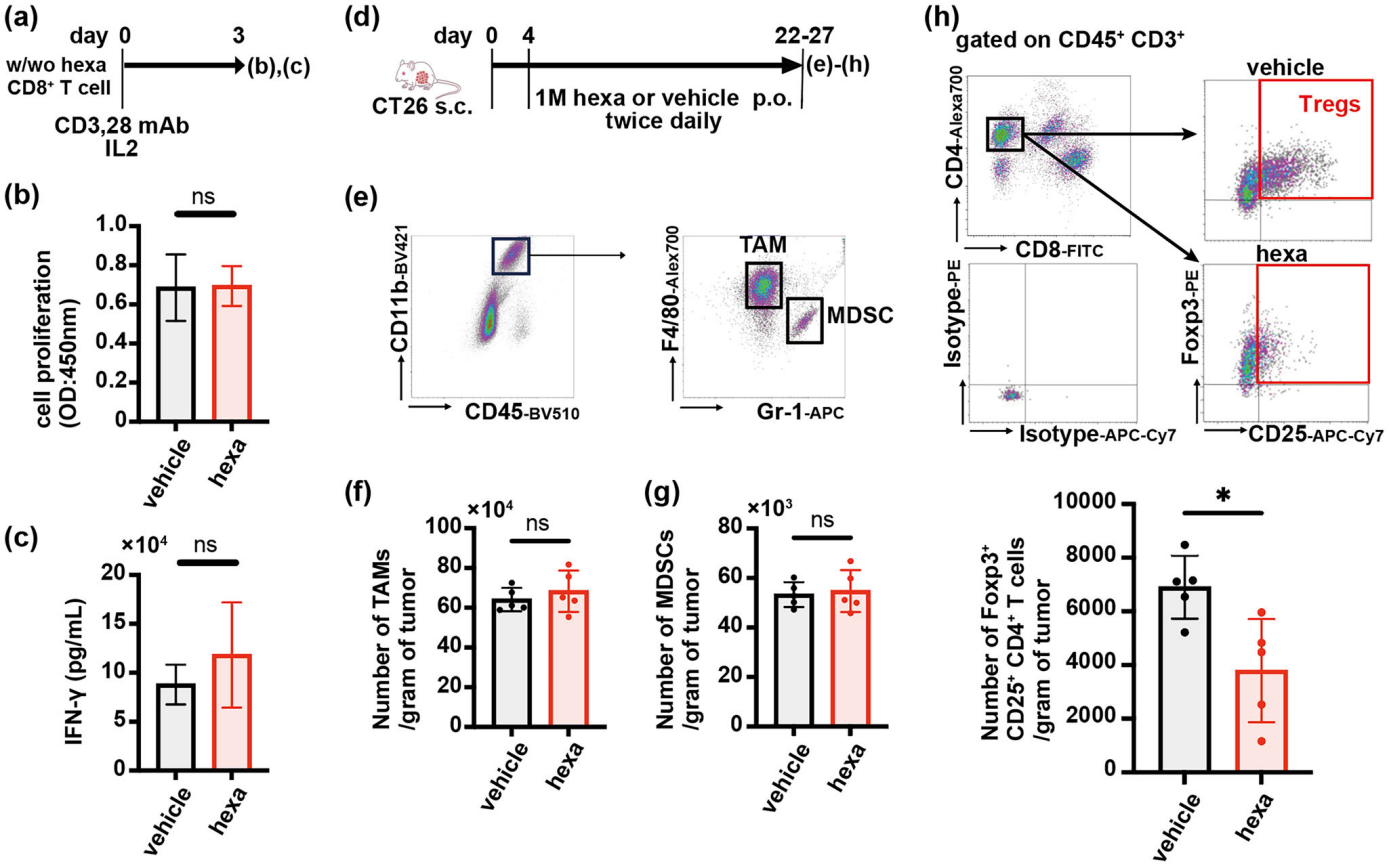
Hexanoate intake suppresses tumor infiltration of Tregs. (a–c) Schematic illustration of the experimental design involving sodium hexanoate (hexa) treatment of CD8^+^ T cells followed by stimulation with anti‐CD3 mAb, anti‐CD28 mAb, and IL‐2 in vitro. (b and c) Effects of culture with/without hexa on the proliferative capacity (b) and IFN‐γ production capacity (c) of CD8^+^ T cells, as evaluated by WST‐1 assay and ELISA, respectively (means ± SD; *n* = 5). (d) Schematic illustration of the experimental design involving subcutaneous flank inoculation of BALB/c mice with 5 × 10^5^ CT26 cells on day 0, followed by treatment with vehicle (200 µL water) or 1 M hexa (equivalent 2760 mg/kg/day) starting on day 4. (e–h) Flow cytometry gating strategies for TAMs (CD45^+^CD11b^+^F4/80^+^Gr‐1^−^) and MDSCs (CD45^+^CD11b^+^F4/80^−^Gr‐1^+^) (e) and analyses of the numbers of TAMs (f), MDSCs (g), and Tregs (CD45^+^CD3^+^CD4^+^CD25^+^Foxp3^+^), showing staining of representative Tregs in each group (upper 4 panels) and graphs of all individuals (lower panel) (h), in the CT26 tumors (means ± SD; *n* = 5). Tumors were excised on days 22–27. Values are expressed as mean ± SD of different mice per group. *p < 0.05, unpaired two‐tailed Student's *t*‐tests. n.s., not significant.

To clarify the mechanism by which hexanoate enhances anti‐tumor immune responses, we evaluated its effects on CD8^+^ T cells in vitro. Hexanoate did not affect the proliferation or IFN‐γ production of mouse CD8^+^ T cells activated with anti‐CD3 and anti‐CD28 mAbs (Figure 3a,b), suggesting that hexanoic acid does not act directly on CD8^+^ T cells. Hypothesizing that hexanoic acid indirectly enhances the anti‐tumor activity of CD8^+^ T cells through immune cells with immunosuppressive activity, we evaluated its effects on TAMs, MDSCs, and Tregs in vivo. Interestingly, hexanoate treatment did not affect the numbers of TAMs or MDSCs in tumors (Figures 3c–e and S5a,b), but markedly suppressed intratumoral infiltration of Tregs (Figure 3f). These results suggested that hexanoic acid indirectly enhances the anti‐tumor activity of CD8^+^ T cells through Tregs.

### 3.3 Hexanoate Acts Directly on Tregs to Reduce Their Immunosuppressive Activity

**FIGURE 4:**
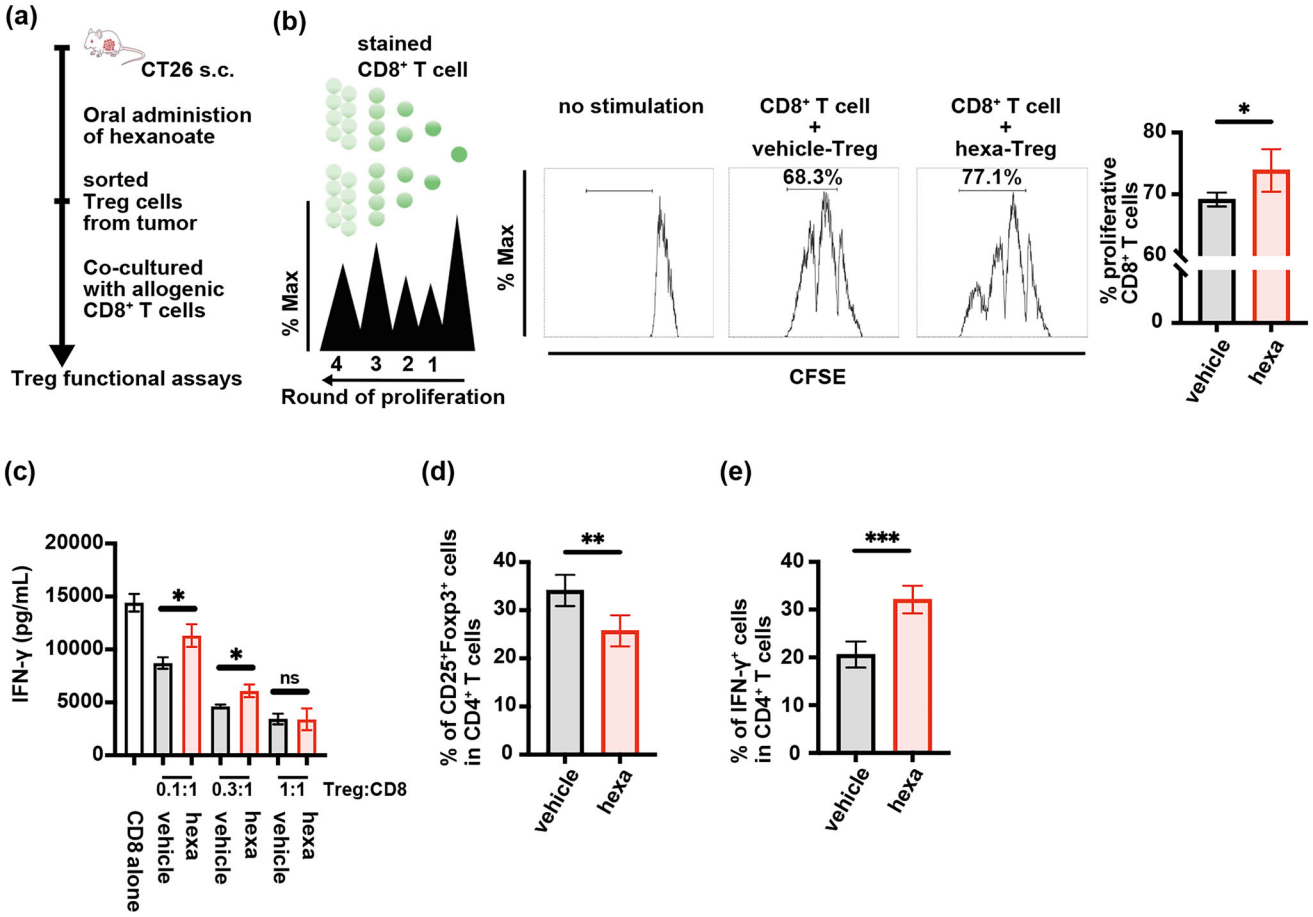
Hexanoate acts directly on Tregs, reducing immunosuppressive activity against CD8^+^ T cells. (a) Schematic illustration of the experimental design of functional assays of Tregs isolated from CT26 tumors of BALB/c mice treated with 1 M hexanoate (hexa; equivalent to 2760 mg/kg/day) or vehicle, then co‐cultured with CD8^+^ T cells derived from splenocytes cultured in the presence of anti‐CD3 mAb, anti‐CD28 mAb, and IL‐2. (b) Representative CFSE staining images (left 3 panels) and graphs of the proliferation of CFSE‐labeled CD8^+^ T cells (1 × 10^5^) co‐cultured with Tregs (1 × 10^4^) from all individual mice (right panel). Proliferation of CD8^+^ T cells was determined by CFSE dilution (means ± SD; *n* = 5). (c) ELISA measurements of IFN‐γ concentrations in the culture supernatants of Tregs and CD8^+^ T cells co‐cultured at the indicated ratios (means ± SD; *n* = 5). (d and e) Percentages of Tregs (d) and Th1 cells (e) differentiated from naïve CD4^+^ T cells isolated from mouse splenocytes (means ± SD; *n* = 5). Values are expressed as mean ± SD of different mice per group. *p < 0.05, **p < 0.01, unpaired two‐tailed Student's *t*‐tests. n.s., not significant.

To elucidate the effects of hexanoate on tumor‐infiltrating Tregs, we isolated hexa‐Tregs and vehi‐Tregs from mouse tumors using MACS beads. These Tregs were then co‐cultured with CFSE‐labeled mouse CD8^+^ T cells derived from splenocytes cultured in the presence of anti‐CD3 mAb, anti‐CD28 mAb, and IL‐2 (Figure 4a). Compared with vehi‐Tregs, co‐culture with a series of hexa‐Treg ratios did not significantly suppress IFN‐γ production or proliferation of the CD8^+^ T cells (Figure 4b,c). Collectively, these findings indicated that hexanoate may act as a negative regulator of Treg‐mediated immunosuppression of CD8^+^ T cells. Subsequently, we treated naïve CD4^+^ T cells isolated from splenocytes with hexanoate, which significantly inhibited their differentiation into Tregs (CD25^+^Foxp3^+^CD4^+^) and promoted Th1 differentiation (IFN‐γ^+^CD4^+^) in vitro (Figures 4d and S6). These findings suggested that hexanoate improves the immunosuppressive TME by inhibiting both the function of Tregs and the differentiation of naïve CD4^+^ T cells into Tregs.

## 4 Discussion

A recent systematic review and meta‐analysis summarizing 31 prospective cohort studies showed a consistent inverse correlation between high dairy and milk intake and CRC risk [27]. However, the mechanism by which milk components may provide therapeutic benefits against CRC is not understood, and there has been no detailed analysis of what components of milk act as protective factors against CRC. In this study, we demonstrated that intake of hexanoate, a form of SCFA/MCFA characteristically found in milk, enhances cancer antigen‐specific T cell responses and suppresses CRC growth in a CT26 tumor‐bearing mouse model. Furthermore, we found that the mechanism by which hexanoate enhances the anti‐tumor immune response involves quashing the Treg‐mediated immunosuppression of CD8^+^ T cells, thereby indirectly enhancing the intratumoral infiltration and effector function of CD8^+^ T cells.

SCFAs/MCFAs reportedly influence the immune system [28, 29], with butyric acid, propionic acid, and acetic acid enhancing the number and function of Tregs in the intestine [30] while exerting anti‐inflammatory effects in inflammatory bowel disease models [31, 32]. Furthermore, these SCFAs/MCFAs exhibit tissue‐specific accumulation, with butyric acid primarily accumulating in the intestine and propionic acid in the liver [33]. By contrast, we found here that hexanoic acid circulated systemically and accumulated at high concentrations in tumor tissues and tumor‐draining lymph nodes. Additionally, it was revealed that hexanoate has the ability to enhance anti‐tumor immune responses in tumor tissues and tumor‐draining lymph nodes through at least three different effects on Tregs. First, hexanoate reduced the differentiation of naïve CD4^+^ T cells into Tregs while promoting the differentiation of naive CD4^+^ T cells into Th1 cells in vitro. Consistent with these findings, Haghikia et al. reported that hexanoic acid promotes the differentiation of naive CD4^+^ T cells into Th1 and Th17 cells via activation of the p38 MAPK signaling pathway [21]. Taken together with the increased hexanoic acid concentration in tumor‐draining lymph nodes following hexanoate intake, these results strongly suggest that hexanoic acid exerts its effects during the priming phase of Th0 differentiation into other CD4^+^ T cell subtypes. Consistent with this, we observed that naïve CD4^+^ T cells were abundant in tumor‐draining lymph nodes but largely absent from tumors (Figure S7). Second, hexanoate intake reduced the immunosuppressive function of mature Tregs against CD8^+^ T cells. Notably, the intratumoral concentration of hexanoic acid increased, suggesting that it also positively impacts the cancer‐immunity cycle in the effector phase. Third, hexanoate significantly inhibited tumor infiltration by Tregs. Although the molecular mechanism of this inhibitory effect is unclear, previous reports have associated hexanoic acid with the regulation of cytokines and chemokines [34]; thus, hexanoic acid may decrease the production of chemokines that recruit Tregs into tumors, namely CCL22 and CCL28. Collectively, these findings suggest that different SCFAs/MCFAs exert distinct immunomodulatory effects, and their therapeutic efficacy depends on their site of accumulation. Therefore, controlling both the quality and quantity of SCFAs/MCFAs in the TME may be crucial for enhancing anti‐tumor immune responses.

Although immune checkpoint inhibitor (ICI) therapy has brought about a paradigm shift in cancer treatment [35, 36, 37], the efficacy of monotherapy is limited and necessitates the development of combination therapy [38]. Attempts have been made to augment the therapeutic effect of anti‐PD‐1 antibodies with various drugs and dietary combinations [25, 39, 40, 41, 42, 43]. While a recent study reported that a mixture of butyric, valeric, and hexanoic acids enhanced the efficacy of anti‐PD‐1 antibodies [44], our findings demonstrated that hexanoate alone was sufficient to potentiate this therapeutic effect. These results suggest that hexanoic acid intake may be an attractive strategy for enhancing the therapeutic effects of ICIs. Additionally, hexanoic acid may improve the efficacy of adoptive cell therapies (ACTs), such as chimeric antigen receptor T‐cell (CAR‐T) and TIL approaches. The major limitation of CAR‐T therapy in solid tumors is poor infiltration of the administered T cells into tumor sites, largely due to the immunosuppressive TME [45, 46, 47]. Our study demonstrated that hexanoate attenuates the immunosuppressive TME through multiple actions on Tregs, highlighting its potential as a therapeutic strategy to overcome this challenge. Indeed, recent studies showed that the SCFAs acetate and pentanoate enhanced the therapeutic efficacy of CAR‐T therapy by promoting the differentiation of naive CD4^+^ T cells into Th17 cells [48]. Additionally, hexanoic acid concentration at the tumor site may have potential as a biomarker for patient selection and treatment optimization in cancer immunotherapy, including ICIs and ACTs such as CAR‐T therapy.

Several limitations of this study should be acknowledged. First, our findings are based on a single tumor model using CT26 CRC cells in BALB/c mice. The generalizability of these results to other CRC subtypes, genetic backgrounds, or cancer types remains to be validated in additional preclinical models. Second, because treatment was initiated after tumor establishment (day 4 post‐inoculation) and enhanced anti‐tumor immune responses were observed in the presence of existing cancer, our study demonstrates a therapeutic rather than preventive effect of hexanoate. Future studies investigating the preventive effects of hexanoate will require distinct experimental designs, such as pre‐treatment protocols or long‐term dietary interventions. Third, the hexanoate dose used in this study (2760 mg/kg/day) translates to ∼165.6 g/day for a 60‐kg human, which may be challenging to achieve through conventional dietary intake. However, given the water solubility of sodium hexanoate, hexanoic acid‐enriched milk products could be developed, supporting its potential as a short‐term dietary supplement during CRC treatment. This warrants further investigation into optimal formulations and delivery methods. Fourth, our study focused on short‐term treatment effects. Comprehensive evaluation of long‐term safety, sustained efficacy, and potential adverse effects of prolonged hexanoate administration is essential before clinical translation can be considered.

Until recently, our understanding of the links between milk and dairy product consumption and cancer risk was mainly based on epidemiological evidence. Given the compositional diversity of the many varieties of milk (e.g., full‐fat, skimmed, and ingredient‐controlled), research on the effectiveness of milk consumption in cancer therapy should focus on identifying individual bioactive ingredients. We anticipate that the findings of this study will contribute to elucidating the molecular mechanisms by which milk components modulate therapeutic immune responses against CRC. Furthermore, this study underscores the potential of dietary interventions, which are generally regarded as therapeutically complementary, to act as direct immunotherapeutic anti‐tumor agents, thus broadening their scope in cancer treatment.

## Conflicts of Interest

The authors declare no conflicts of interest.

## Ethics Statement

All animal studies were reviewed and approved by the Nihon University Institutional Animal Care and Use Committee (approval number: AP22MED054‐1 and AP23MED053‐1).

## Supporting information




**Supporting Information file 1**: mnfr70229‐sup‐0001‐SuppMat.pdf

**Supporting Information file 2**: mnfr70229‐sup‐0002‐SuppMat.docx

## Data Availability

All data relevant to the study are included in the article or as online supporting information.
