# Supplementary material for: Hexanoic Acid Intake Enhances Anti‐Tumor Immune Responses in Colorectal Cancer by Reducing the Immunosuppressive Function of Tregs
Source: Mol Nutr Food Res. 2025 Aug 23;69(22):e70229. doi: 10.1002/mnfr.70229 (PMC12643187; doi:10.1002/mnfr.70229)
Supplement: Supplementary file 1 — Supporting Information file 1: mnfr70229‐sup‐0001‐SuppMat.pdf [file MNFR-69-e70229-s001.pdf]

Figure S1

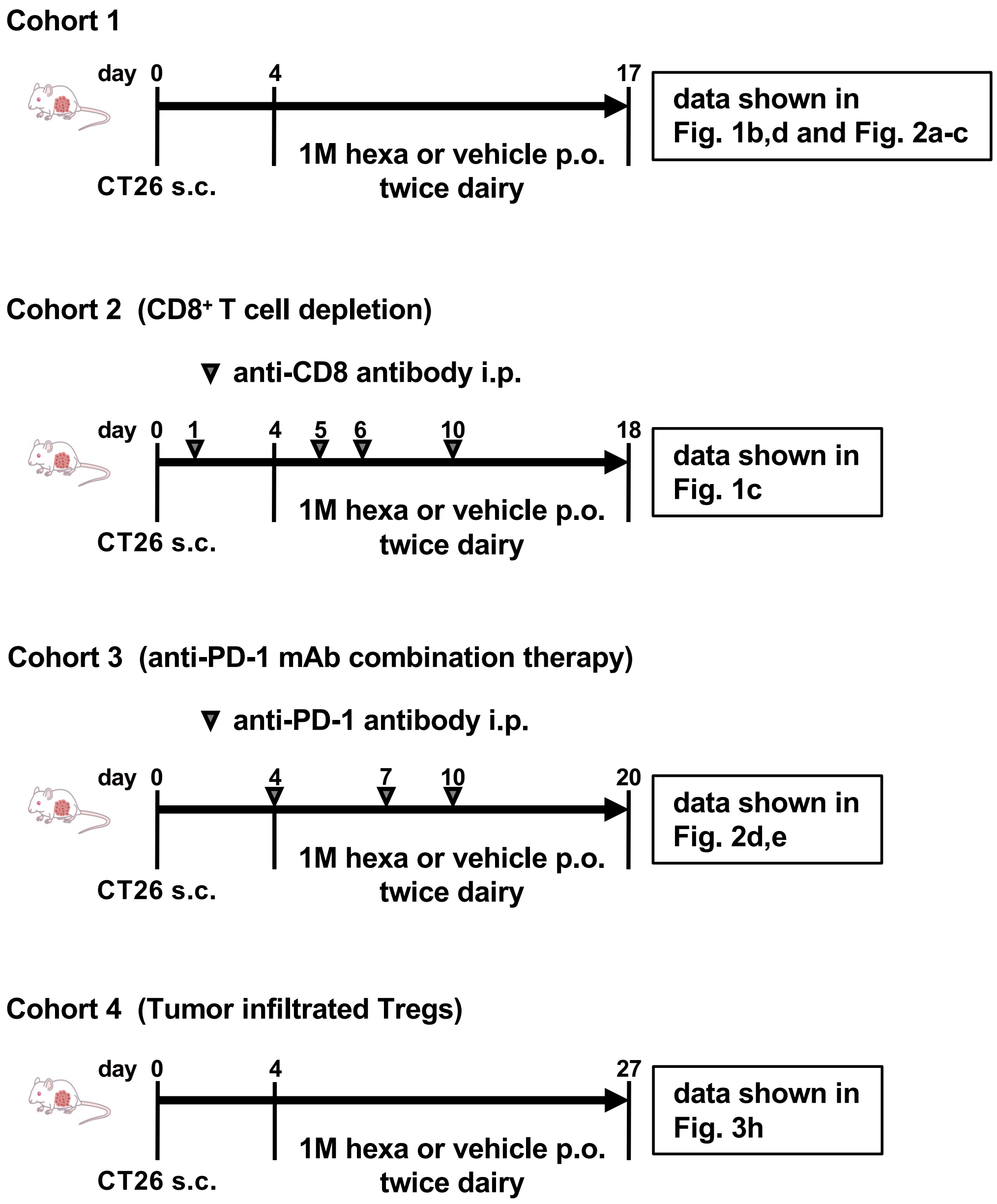
Cohort 1
day 0
4
17
CT26 s.c.
1M hexa or vehicle p.o.
twice dairy
data shown in
Fig. 1b,d and Fig. 2a-c
Cohort 2 (CD8+ T cell depletion)
▼ anti-CD8 antibody i.p.
day 0
1
4
5
6
10
18
CT26 s.c.
1M hexa or vehicle p.o.
twice dairy
data shown in
Fig. 1c
Cohort 3 (anti-PD-1 mAb combination therapy)
▼ anti-PD-1 antibody i.p.
day 0
4
7
10
20
CT26 s.c.
1M hexa or vehicle p.o.
twice dairy
data shown in
Fig. 2d,e
Cohort 4 (Tumor infiltrated Tregs)
day 0
4
27
CT26 s.c.
1M hexa or vehicle p.o.
twice dairy
data shown in
Fig. 3h

Figure S2

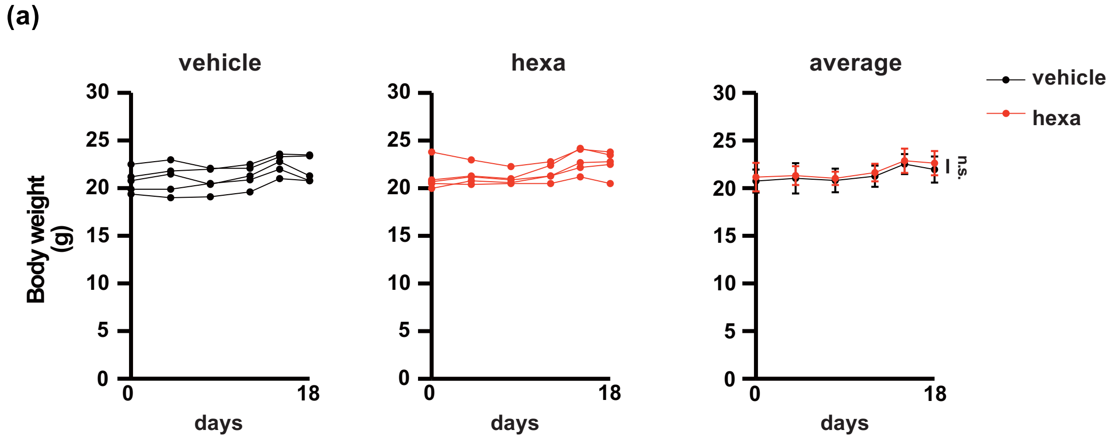

(a)
vehicle
hexa
average
Body weight (g)
30
25
20
15
10
5
0
0
18
days
n.s.
vehicle
hexa

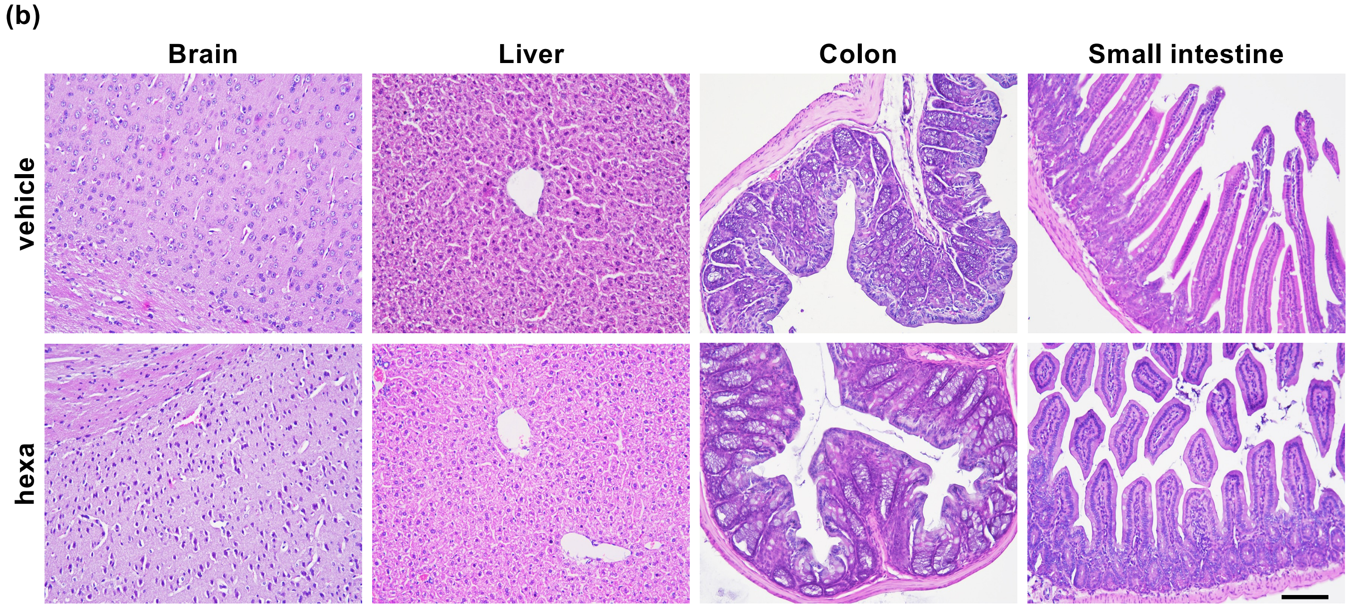

(b)
Brain
Liver
Colon
Small intestine
vehicle
hexa

Figure S3

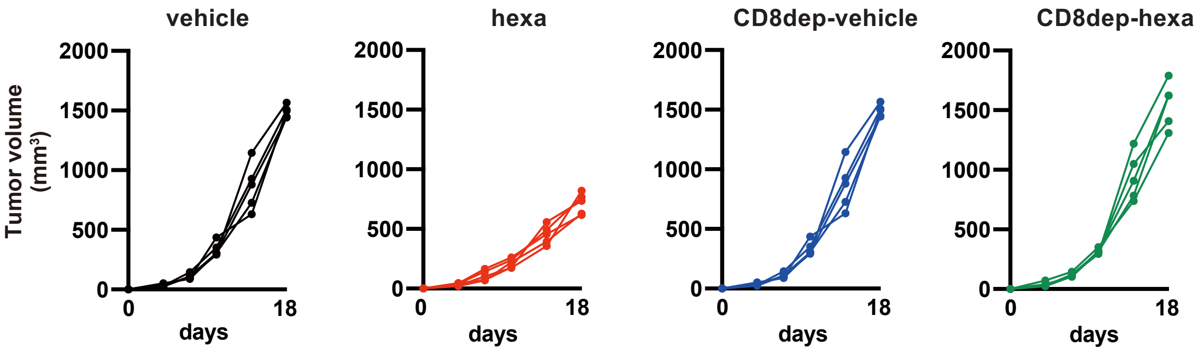

vehicle
hexa
CD8dep-vehicle
CD8dep-hexa
Tumor volume
(mm³)
2000
1500
1000
500
0
0
18
days

Figure S4

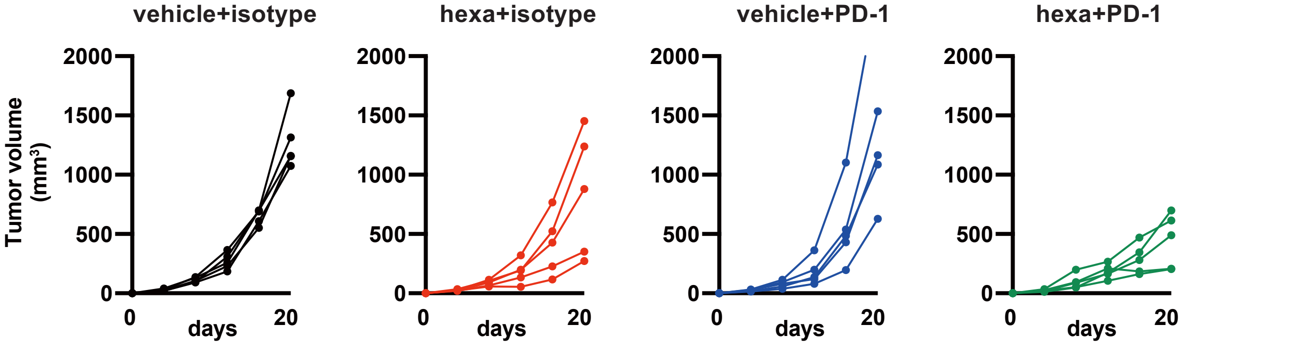

vehicle+isotype
hexa+isotype
vehicle+PD-1
hexa+PD-1
Tumor volume
(mm³)
2000
1500
1000
500
0
0
days
20

Figure S5

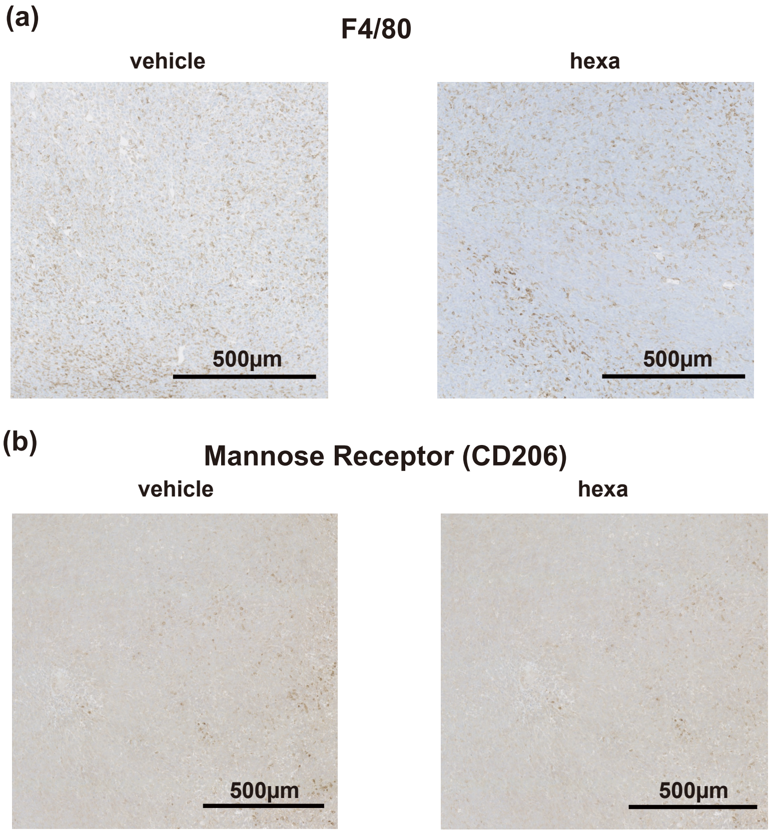
(a)
F4/80
vehicle
hexa
500μm
500μm
(b)
Mannose Receptor (CD206)
vehicle
hexa
500μm
500μm

Figure S6

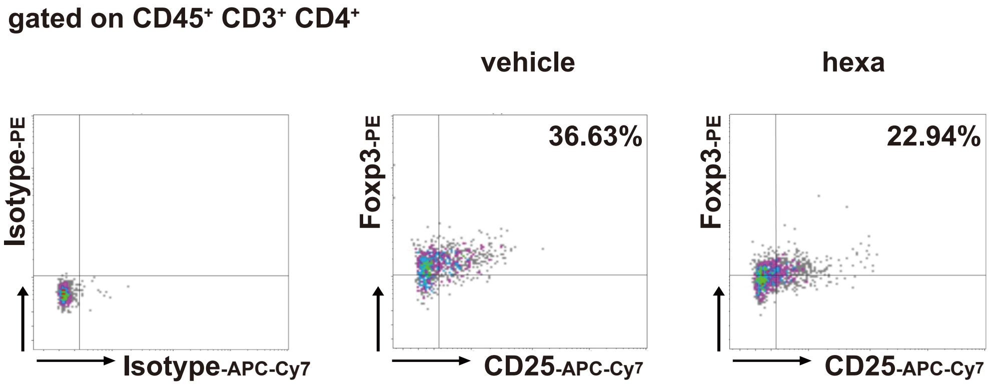

gated on CD45+ CD3+ CD4+
vehicle
hexa
Isotype-PE
Isotype-APC-Cy7
Foxp3-PE
36.63%
CD25-APC-Cy7
Foxp3-PE
22.94%
CD25-APC-Cy7

Figure S7

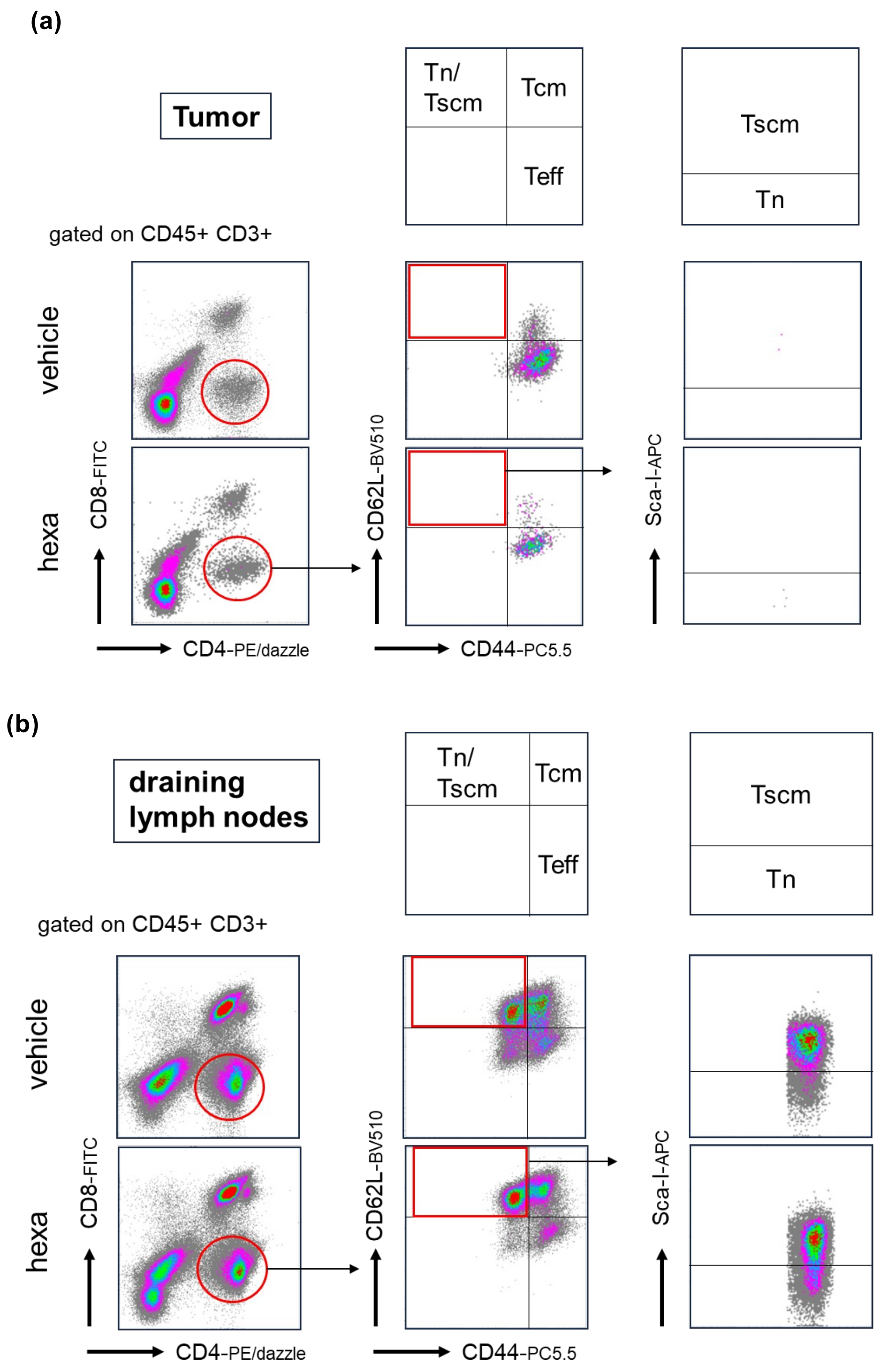
(a)
Tumor
Tn/
Tscm
Tcm
Teff
Tscm
Tn
gated on CD45+ CD3+
vehicle
hexa
CD8-FITC
CD4-PE/dazzle
CD62L-BV510
CD44-PC5.5
Sca-I-APC
(b)
draining
lymph nodes
Tn/
Tscm
Tcm
Teff
Tscm
Tn
gated on CD45+ CD3+
vehicle
hexa
CD8-FITC
CD4-PE/dazzle
CD62L-BV510
CD44-PC5.5
Sca-I-APC
