# Supplementary material for: Hexanoic Acid Intake Enhances Anti‐Tumor Immune Responses in Colorectal Cancer by Reducing the Immunosuppressive Function of Tregs
Source: Mol Nutr Food Res. 2025 Aug 23;69(22):e70229. doi: 10.1002/mnfr.70229 (PMC12643187; doi:10.1002/mnfr.70229)
Supplement: Supplementary file 2 — Supporting Information file 2: mnfr70229‐sup‐0002‐SuppMat.docx [file MNFR-69-e70229-s002.docx]

**Figure. S1: Summary of the mouse cohorts used in each experiment.** Figures 1b, 1d, and 2a–c were all derived from cohort 1, while Figures 1c, 2d and 2e, and 3h were derived from cohorts 2, 3 and 4, respectively. A reduction in tumor volume and an increase in CD8^+^ T-cell infiltration in the hexanoate treatment group compared with the findings in the vehicle group was confirmed for all experimental cohorts.

**Figure. S2: Hexanoate administration has no obvious adverse effects on normal tissues in vivo.** CT26 tumor-bearing BALB/c mice were administered 1M hexanoate or vehicle only (vehicle), and their body weights were monitored. **(a)** Individual body weight growth curves (left two panels) and group average body weights (g ± SD; n = 5) (right panel). **(b)** Representative HE-stained sections of normal liver, small intestine, colon, and brain tissues. Scale bar: 100 μm.

**Figure S3: Hexanoate-mediated tumor growth inhibition is abrogated by CD8^+^ T-cell depletion.** Individual tumor volumes from vehicle- and hexanoate (hexa)-treated mice that received CD8-depleting or isotype-matched mAbs. Also see related Figure 1c.

**Figure S4: Systemic administration of hexanoate enhances the therapeutic effect of anti-PD-1 antibodies.** Individual tumor volumes in vehicle- and hexanoate (hexa)-treated mice that received anti-PD-1 or isotype-matched antibodies (200 µg/mouse). Also see related Figure 2d.

**Figure S5: Hexanoate intake has no effect on tumor-infiltrating macrophages. (a, b)** Immunostaining of macrophage marker molecules F4/80 **(a)** and mannose receptor (CD206) **(b)** in CT26 tumors from BALB/c mice treated with 1 M hexanoate (hexa) or vehicle only (vehicle).

**Figure S6: Hexanoate inhibits differentiation of naïve CD4^+^ T cells into Tregs.** Gating strategy for Tregs (CD45^+^CD4^+^CD25^+^Foxp3^+^) and staining of representative Tregs in the hexanoate and vehicle treatment groups of mice. Also see related Figure 4d.

**Figure S7: Naive CD4^+^ T cells are abundant in tumor-draining lymph nodes but not in tumors.** CT26 tumor-bearing BALB/c mice were treated with 1M hexanoate (hexa) or vehicle only (vehicle). **(a, b)** Flow cytometric analysis of naïve, stem cell memory, central memory, and effector CD4^+^ T cells within tumors **(a)** and tumor-draining lymph nodes **(b)**. The CD4^+^ T-cell subsets were defined as follows: naïve (Tn), CD62L^+^CD44^-^Sca-1^-^, stem cell memory (Tscm), CD62L^+^CD44^-^Sca-1^+^; central memory (Tcm), CD62L^+^CD44^+^; and effector (Teff), CD62L^-^CD44^+^.
